# Complex interactions can create persistent fluctuations in high-diversity ecosystems

**DOI:** 10.1371/journal.pcbi.1007827

**Published:** 2020-05-15

**Authors:** Felix Roy, Matthieu Barbier, Giulio Biroli, Guy Bunin

**Affiliations:** 1 Institut de physique théorique, Université Paris Saclay, CEA, CNRS, Gif-sur-Yvette, France; 2 Laboratoire de Physique de l’Ecole Normale Superieure, ENS, Université PSL, CNRS, Sorbonne Université, Université Paris-Diderot, Sorbonne Paris Cité, Paris, France; 3 Centre for Biodiversity Theory and Modelling, Theoretical and Experimental Ecology Station, CNRS and Paul Sabatier University, Moulis, France; 4 Department of Physics, Technion-Israel Institute of Technology, Haifa, Israel; Abdus Salam International Centre for Theoretical Physics, ITALY

## Abstract

When can ecological interactions drive an entire ecosystem into a persistent non-equilibrium state, where many species populations fluctuate without going to extinction? We show that high-diversity spatially heterogeneous systems can exhibit chaotic dynamics which persist for extremely long times. We develop a theoretical framework, based on dynamical mean-field theory, to quantify the conditions under which these fluctuating states exist, and predict their properties. We uncover parallels with the persistence of externally-perturbed ecosystems, such as the role of perturbation strength, synchrony and correlation time. But uniquely to endogenous fluctuations, these properties arise from the species dynamics themselves, creating feedback loops between perturbation and response. A key result is that fluctuation amplitude and species diversity are tightly linked: in particular, fluctuations enable dramatically more species to coexist than at equilibrium in the very same system. Our findings highlight crucial differences between well-mixed and spatially-extended systems, with implications for experiments and their ability to reproduce natural dynamics. They shed light on the maintenance of biodiversity, and the strength and synchrony of fluctuations observed in natural systems.

## Author summary

Large abundance fluctuations are well-documented in natural populations. Yet, it is still not known to what extent these fluctuations stem from multi-species interactions, rather than environmental perturbations or demographic processes. There have been long-standing debates on these issues, questioning even the possibility of interaction-driven fluctuations, as they might induce species extinctions until equilibrium is reached.

The situation is all the more challenging and richer in complex high-dimensional settings (many interacting species, many niches, etc.), which feature qualitatively new phenomena, and where theory is still lacking. Here we show that high-diversity metacommunities can persist in dynamically-fluctuating states for extremely long periods of time without extinctions, and with a diversity well above that attained at equilibrium. We describe the quantitative conditions for these endogenous fluctuations, and the key fingerprints which would distinguish them from external perturbations.

We establish a theoretical framework for the many-species dynamics, derived from statistical physics of out-of-equilibrium systems. These settings present unique challenges, and observed behaviors may be counter-intuitive, making specialized theoretical techniques an indispensable tool. Our theory exactly maps the many-species problem to that of a single representative species (metapopulation). This allows us to draw connections with existing theory on perturbed metapopulations, while accounting for unique properties of endogenous feedbacks at high diversity.

## Introduction

While large temporal variations are widespread in natural populations [1, 2], it is difficult to ascertain how much they are caused by external perturbations, or by the ecosystem’s internal dynamics, see e.g. [3, 4]. In particular, both theoretical tools and empirical results come short of addressing a fundamental question: can we identify when fluctuations in species abundances arise from complex ecological interactions?

Our focus here is on high-diversity communities. Historically, studies of endogenous fluctuations have focused on single populations or few species [5–7]. On the other hand, theories of many-species interaction networks often center on ecosystems that return to equilibrium in the absence of perturbations [8]. Some authors have even proposed that fluctuations driven by interactions are generally too rare or short-lived to matter, since they can be self-defeating: dynamics that create large erratic variations lead to extinctions, leaving only species whose interactions are less destabilizing, until an equilibrium is reached [9, 10]. Here we go past both the equilibrium [8, 11] or few-species starting points [5–7], to look directly at the dynamics of high-diversity communities in a spatially extended systems.

**Fig 1:**
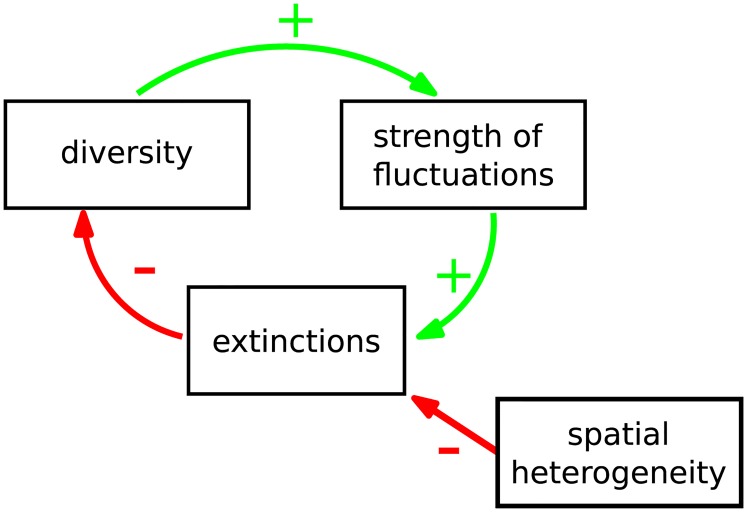
(Top) The fluctuation-diversity feedback cycle. Species diversity is required to maintain endogenous fluctuations. But these fluctuations cause extinctions, which reduce diversity. This negative feedback cycle can lead to the disappearance of endogenous fluctuations, especially in a well-mixed community. However, if spatial heterogeneity can limit extinctions, this negative feedback loop may slow down and create a fluctuating state that persists for very long times.

Many-species endogenous fluctuations can only persist if they do not induce too many extinctions (Fig 1). Extinction rates depend critically on the amplitude of fluctuations [12, 13], their synchrony [14] and their correlation time [15]. The peculiarity of endogenous fluctuations is that these properties arise from the species dynamics, and therefore feed back on themselves. A theory of these feedbacks is however lacking.

Here we propose a novel quantitative approach, and show that many-species endogenous fluctuations can persist for extremely long times. Furthermore, they can be realized in experimental conditions, and identified in these experiments by multiple characteristic features. Crucially, we show that states with higher species diversity have stronger fluctuations, and vice versa. We also offer reasons why they may not have been observed in previous studies, and directions in which to search. An important factor in maintaining a dynamically fluctuating state is the spatial extension of the ecosystem, here modeled as a metacommunity: multiple patches (locations in space) that are coupled by migration.

While equilibria are bound by linear stability, beyond that diversity there exist dynamically fluctuating states, with abundance fluctuations that grow continuously with the diversity. This places equilibria within a broader continuum which also includes non-equilibrium states. And as equilibria at high diversity have a unique phenomenology and require dedicated tools [8, 11], so do these high-diversity, dynamically fluctuating states.

Our strategy is the following. We first propose and simulate experiments to show that persistent fluctuations can be very elusive in a single well-mixed community, yet attainable in a metacommunity via three main ingredients: the existence of multiple patches, moderate migration fluxes coupling them, and differences in conditions between patches. These three ingredients can dramatically reduce the likelihood that large fluctuations within a patch will lead to overall extinctions (see Fig 1), and make it possible for species to persist in highly fluctuating states. We then offer a quantitative understanding of this phenomenon. We build on the analytical framework developed in [16] (dynamical mean-field theory, see also [17–19]) that allows us to investigate, in a quantitative and predictive way, the conditions under which robust fluctuations can arise from complex interactions. This theory exactly maps a deterministic metacommunity (many-species dynamics over multiple spatial locations) to a *stochastic representative metapopulation* (single-species dynamics over multiple spatial locations). It predicts the distribution of abundance, survival and variability for a species subjected to “noise” that results from other species in the same community, rather than external perturbations. Dynamical mean-field theory allows us to analyze these fluctuations, and show that the effective stochasticity of species dynamics is a manifestation of high-dimensional chaos.

The intuitive picture that emerges from our analysis is the following: the persistence of endogenous fluctuations, which can be found in a wide range of realistic conditions, requires a balancing act between forces that stabilize and destabilize the dynamics, see Fig 1. On the one hand, the system needs to preserve a high diversity (both in terms of species number and interaction heterogeneity), as it is known [8, 11] that lower diversity leads to a stable equilibrium. On the other hand, the system also has to limit excursions towards very low abundances. This requires weeding out species that induce unsustainable fluctuations, and rescuing the others from sudden drops.

To accomplish that, the system relies on asynchronous dynamics between different spatial locations, and finite strength and correlation time of the abundance fluctuations. Even though all species show large fluctuations (so that interactions in a patch often switch between being favorable and unfavorable to a given species), long-lasting “sources” emerge for some of the species, i.e. patches where these species are, on average, more likely to remain away from extinction. Rare dynamical fluctuations leading to extinction in a given patch are hampered by migration from the other patches, which keeps the system in a non-equilibrium state. We show that, with moderate migration and some spatial heterogeneity, high-diversity dynamical states can be reached where species populations fluctuate over orders of magnitude, yet remain bounded for very long times above their extinction threshold.

In a parallel work, Pearce et. al. [19] study a different model, in the limit of strictly antisymmetric interactions, generalizing the classic Lotka-Volterra predator-prey cycles to many species, and also find dynamically persistent fluctuations.

Our findings allow us to paint a more precise picture of when persistent endogenous fluctuations can arise. We conclude with a discussion of the implications for biodiversity and ecosystem stability, and predictions for future experiments on community dynamics.

## Results and methods

### Proposed experiments

**Fig 2:**
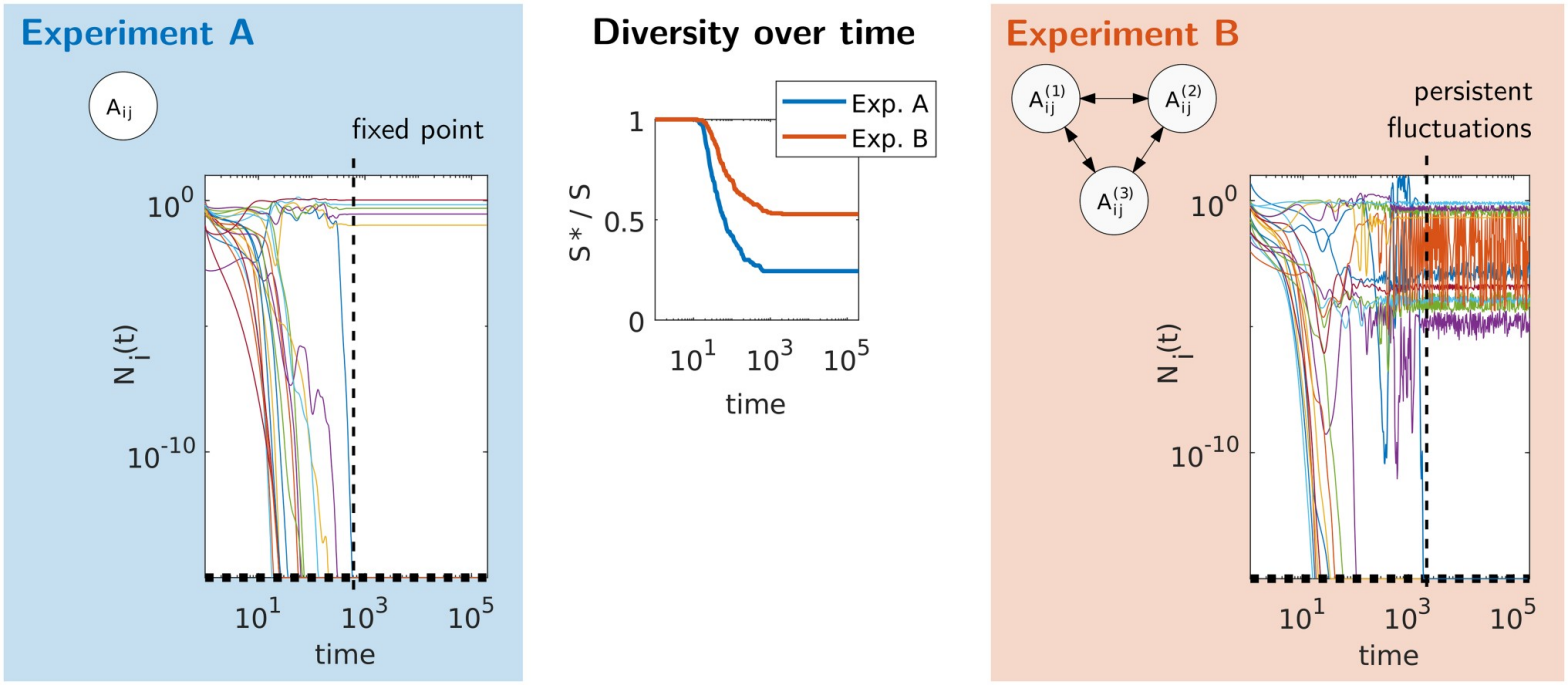
Numerical realization of the proposed experiments, illustrating conditions that lead to a fixed point or persistent fluctuations. (A) A single patch (well-mixed community) with an interaction matrix *A*_*ij*_. (B) Multiple patches connected by migration, with slightly different conditions (e.g. temperature or resources) in each patch, represented here by location-dependent parameters such as *A*_*ij*,*u*_. In the right and left panels we show the time evolution of a few representative species abundances *N*_*i*_(*t*): Experiment A, with a single patch (*M* = 1) reaches a fixed point, while in experiment B a meta-community with *M* = 8 patches reaches a stationary chaotic state (*S* = 250). Middle panel: Fraction of persistent species (S* out of a pool of *S* = 250 species) as a function of time. Parameters and values for running the simulations are given in S1 Text (Appendix A).

In the following, we introduce our results via a set of proposed experiments, realized in simulations, see Fig 2. These results are later explained in the theoretical analysis. All parameters for simulations are detailed in S1 Text (Appendix A).

We focus on a meta-community which consists of *M* patches (well-mixed systems) connected by migration, and isolated from the external world. As an archetype of complex ecological dynamics, we consider generalized Lotka-Volterra equations with random interactions, which have been the focus of many recent theoretical advances [20–22]. The dynamics of the abundance *N*_*i*,*u*_ of species *i* in patch *u* read:

$$\begin{array}{cc} \frac{d}{dt}N_{i,u} & =N_{i,u}[B_{i,u}-N_{i,u}-\sum_{j}A_{ij,u}\;N_{j,u}]\\ & \;\;\;+\sum_{v}D_{i,uv}\;(N_{i,v}-N_{i,u})\;. \end{array}$$ (1)

where *A*_*ij*,*u*_ are the interactions coupling the species, *B*_*i*,*u*_ represents the equilibrium abundance in absence of interactions and migration (known as the carrying capacity), and *D*_*i*,*uv*_ are the migration rates between patches *u* and *v*. In addition, an extinction threshold is implemented as follows: when a species’ abundance goes below a cutoff *N*_*c*_ in *all* patches, the species is removed from the metacommunity and cannot return. This threshold corresponds to the minimum sustainable number of individuals, hence 1/*N*_*c*_ sets the scale for the absolute population size (*P*) of the species. This recipe combines differential equations, which applies when populations are large, while still allowing for extinctions. For simplicity, we take *D*_*i*,*uv*_ = *d*/(*M* − 1) and *N*_*c*_ identical for all species and patches.

The species are assumed to have unstructured interactions (e.g. they belong to the same trophic level), meaning that *A*_*ij*,*u*_ are sampled independently and identically for different (*i*, *j*). (Our results also hold when *A*_*ij*,*u*_ and *A*_*ji*,*u*_ are correlated, see S1 Text (Appendix F).) For a given species pair, its interactions *A*_*ij*,*u*_ vary somewhat with *u*; this variability corresponds to small differences in the conditions between the patches [23]. In the simulation examples we set all carrying capacities *B*_*i*,*u*_ = 1; the phenomena described below are also found if *B*_*i*,*u*_ vary between patches in addition to, or instead of the interaction coefficients.

**Fig 3:**
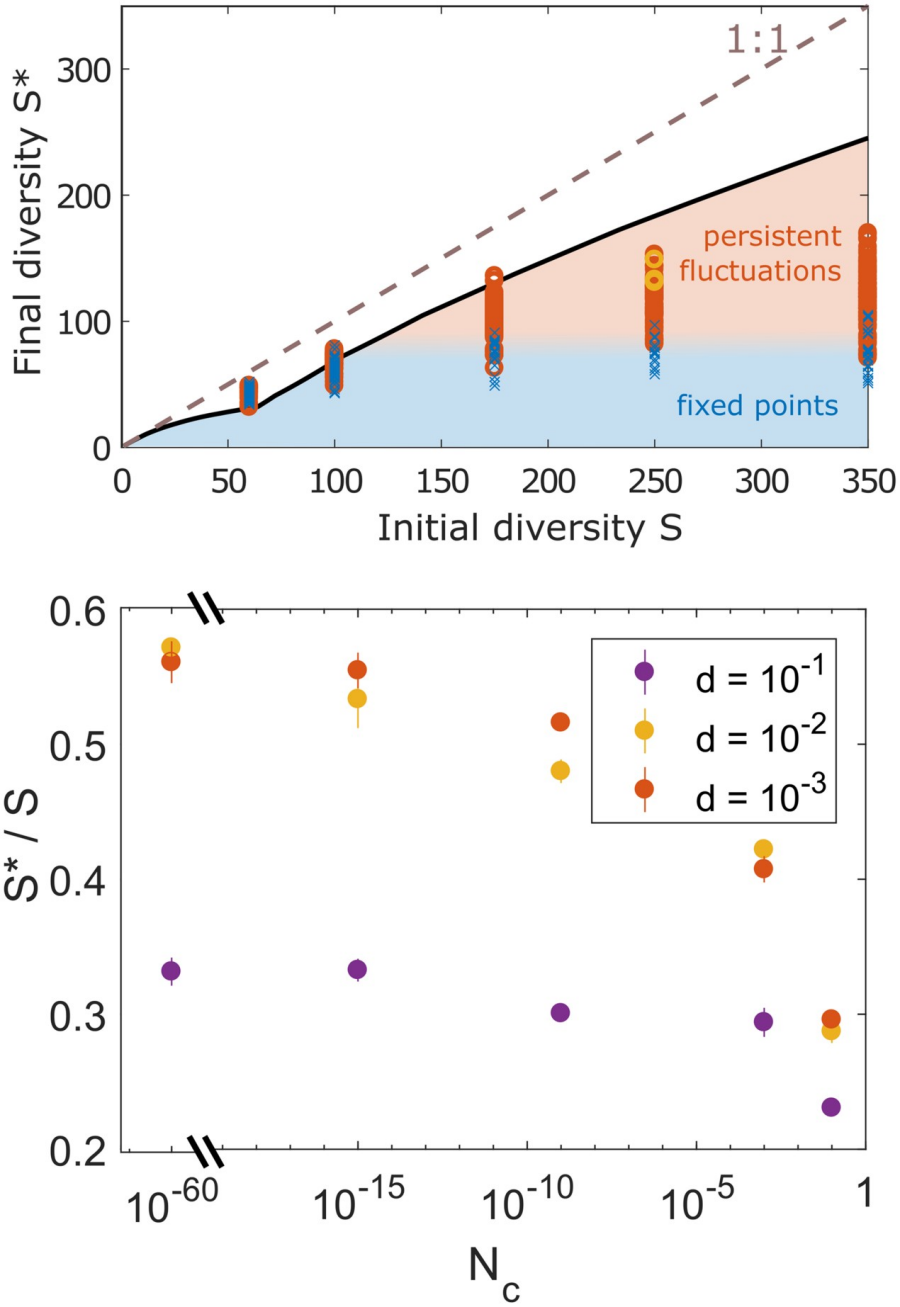
(Top) Species diversity at long times, compared to the theoretical bound obtained in S1 Text (Appendix D) for large *S* (solid line). The bound depends on the distribution of interactions, carrying capacities and initial pool size *S*. Each symbol represents the state at the end of one simulation run, with different values of the migration rate *d* and the abundance cut-off *N*_*c*_, with fluctuating states (circles) and fixed points (crosses). Three yellow circles (two of which are overlaid) show the diversity in three runs with the same conditions as in Fig 2(B), all reaching dynamically-fluctuating states and similar diversities. States closer to the theoretical bound (with higher diversity) also exhibit larger fluctuations and are more difficult to reach due to extinctions in the transient dynamics (see Fig 2). The dashed line represents full survival (S* = *S*). (Bottom) The final diversity is set by the transient dynamics, which is affected by factors such as the migration strength and the total population size (1/*N*_*c*_).

Our proposed experiments, illustrated by dynamical simulations, are the following:

(A) First, we model a single patch, *M* = 1 initially containing *S* = 250 species. Each interaction coefficient is non-zero with probability *c* = 1/8, and the non-zero interactions are Gaussian with mean(*A*_*ij*,*u*_) = 0.3, std(*A*_*ij*,*u*_) = 0.45. Species go extinct until the system relaxes to a fixed point (stable equilibrium), see left panel of Fig 2.
(B) We now take *M* = 8 patches with the same initial diversity *S* = 250 and interaction statistics as in (A). For each pair of interacting species, *A*_*ij*,*u*_ varies slightly with location *u*, with a correlation coefficient *ρ* = 0.95 between patches. The abundances now fluctuate without reaching a fixed point, see right panel of Fig 2. At first the diversity decreases as species go extinct, but this process dramatically slows down, and the diversity is unchanged at times on the order of 10^5^, see middle panel of Fig 2. This result is robustly reproducible: repeating the experiment three times, with interaction and initial conditions sampled anew each time, dynamical fluctuations are reached and at a similar long-time diversity, see Fig 3(top).

Three essential observations emerge from simulating these experiments, and repeating them for different parameters. First, species diversity and the strength of endogenous fluctuations are tightly bound, each contributing to the other’s maintenance. Second, as shown in Fig 2, species trajectories first go through a transient phase where they fluctuate over many orders of magnitude, causing numerous extinctions which lead to a reduction of variability, until a fixed point (for *M* = 1) or non-equilibrium state (for *M* = 8) with weaker fluctuations is reached. Third, the qualitative difference between experiments A and B is robust to changes in parameter values. Changes in *N*_*c*_ and *d* affect only quantitatively the states that are reached in experiment B, see Fig 3(top). For instance, by increasing the population size *P* = 1/*N*_*c*_, we can reach dynamically fluctuating states with higher long-time diversities, as shown in Fig 3(bottom). When the population size is reduced by increasing *N*_*c*_, the long-time diversity decreases, but remains high until *N*_*c*_ ∼ 10^−2^ − 10^−1^, where it decreases dramatically. For example, the diversity shown in Fig 2(right) is 80%_±13%_ higher than that reached for fixed-points with precisely the same number of patches, interactions and migration. (Fixed points were found by removing species after the dynamical state has stabilized, by increasing *N*_*c*_ until a fixed point was obtained.) Similarly, as long as the migration coefficient is in the range *d* ≲ 0.1 the main qualitative results remain unaltered.

### Theory

We now aim to understand which conditions allow a fluctuating state to be reached and maintained without loss of species.

#### Dynamical mean field theory

We build on a powerful theory, known as Dynamical Mean Field Theory (DMFT) that exactly maps the deterministic meta-community problem (many species in multiple patches) to a stochastic meta-population problem (single species in multiple patches). When species traits and interactions are disordered, e.g. drawn at random from some probability distributions, all species can be treated as statistically equivalent [24]. We can then describe the whole system by following the trajectory of a single species, randomly sampled from the community, and studying its statistics. In the DMFT framework, the effect of all other species on that single species is encapsulated by an “ecological noise” term generated by their fluctuations. This is analogous to the use, in physics, of thermal noise to represent interactions between an open system and its environment. Since species are statistically equivalent, the properties of this ecological noise can be self-consistently obtained from the dynamics of the single species.

While the theory applies to all times [16], as discussed in S1 Text (Appendix B), we only consider here the stationary state reached after a long time, in which extinctions are already rare. (We will see in the next section that, when endogenous fluctuations are present, this state is actually metastable, i.e. it is almost stationary on large but finite time-scales.) In that state, observables such as the mean abundance are stable over time, and two-time measures, such as correlation functions between times *t* and *t*′, depend only on the difference *t* − *t*′. This entails that each species abundance fluctuates with a finite correlation time, i.e. it tends to return to some constant characteristic value after a finite time.

The result of this mapping is that the abundance *N*_*u*_ of a given species in patch *u* undergoes stochastic dynamics,

$$\begin{array}{c} \frac{dN_{u}}{dt}=N_{u}(N_{u}^{*}-N_{u}+\xi_{u})+\sum_{v}D_{uv}(N_{v}-N_{u})\;. \end{array}$$

This equation models the dynamics of the target species, including its interactions with other species whose abundances are fluctuating. The contribution of interactions can be separated into a time-independent and a time-dependent parts. The time-independent part goes into $N_{u}^{*}$, the characteristic value around which the species abundance will fluctuate in the patch. It differs between species and between patches, due to interactions and to environmental preferences modeled by *B*_*i*,*u*_ in Eq (1), and follows a multivariate Gaussian distribution. The time-dependent part is encapsulated in *ξ*_*u*_(*t*), a Gaussian noise with a finite correlation time.

As the quantities $N_{u}^{*}$ and *ξ*_*u*_(*t*) result from interaction with other species, which are statistically equivalent to the target species, one can express their properties from the statistics of *N*_*u*_(*t*) itself, as shown in S1 Text (Appendix B). The most important features are that time-dependent Gaussian noise *ξ*_*u*_(*t*) has zero mean and a covariance *C*_*ξ*_(*t*, *t*′), which is directly related to the time auto-correlation *C*_*N*_(*t*, *t*′) of *N*_*u*_(*t*) within a patch, and to *σ*^2^ = *cS* var(*A*_*ij*_) the variance of interactions rescaled as in [8]. Moreover, the covariance of the $N_{u}^{*}$ is fixed by the time auto-correlation of *N*_*u*_(*t*), both within and in-between patches. In principle, the noise is also correlated between patches, but this is a small effect in the dynamical regime of interest to us, see the next section. Note that *C*_*ξ*_(*t*, *t*′) vanishes when a stable equilibrium is reached.

**Fig 4:**
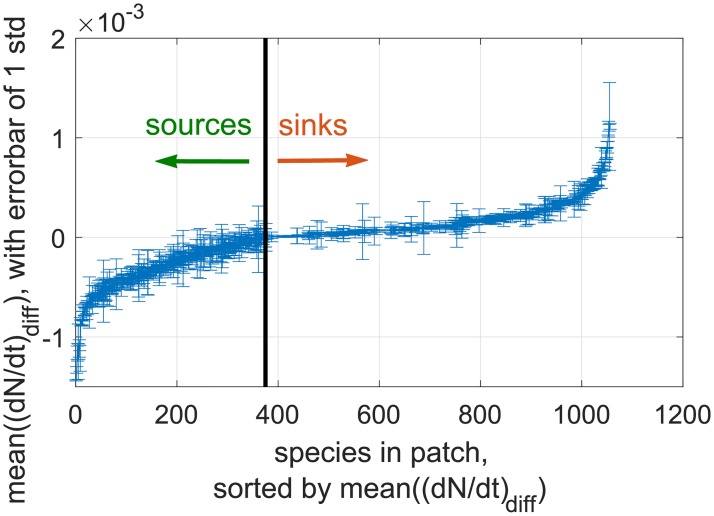
Sources maintain their identity over time. The degree to which a patch is a source for a given species is measured by (*dN*/*dt*)_diff_, the contribution of diffusion to the change of *N*(*t*), which is negative for sources and positive for sinks. We show all species-patch pairs ordered by the average of this quantity over long times, with error bars giving its standard deviation. For 94% of sources, and 85% of all species-patch pairs, this quantity (*dN*/*dt*)_diff_ retains its sign most of the time, being at least one standard deviation away from zero.

The analysis of the DMFT equations clarifies the main effect of coupling patches by migration: patches with higher $N_{u}^{*}$ tend to act as sources, i.e. the species most often grows there, and migrates out to sites where it cannot grow (sinks). We show directly from simulations of the Lotka-Volterra equations in Fig 4 that species have particular patches which tend to act as sources consistently over long times. This fact is counter-intuitive, as the abundances of all species may be fluctuating over orders of magnitude in any given patch, yet this patch will retain its identity as a source (or sink) when averaging over long time periods. The variability of the $N_{u}^{*}\text{s}$ between patches thus leads to an insurance effect, since it is enough to have one patch acting as a source to avoid extinction of the species in the others.

#### Reaching and maintaining a dynamical state

**Fig 5:**
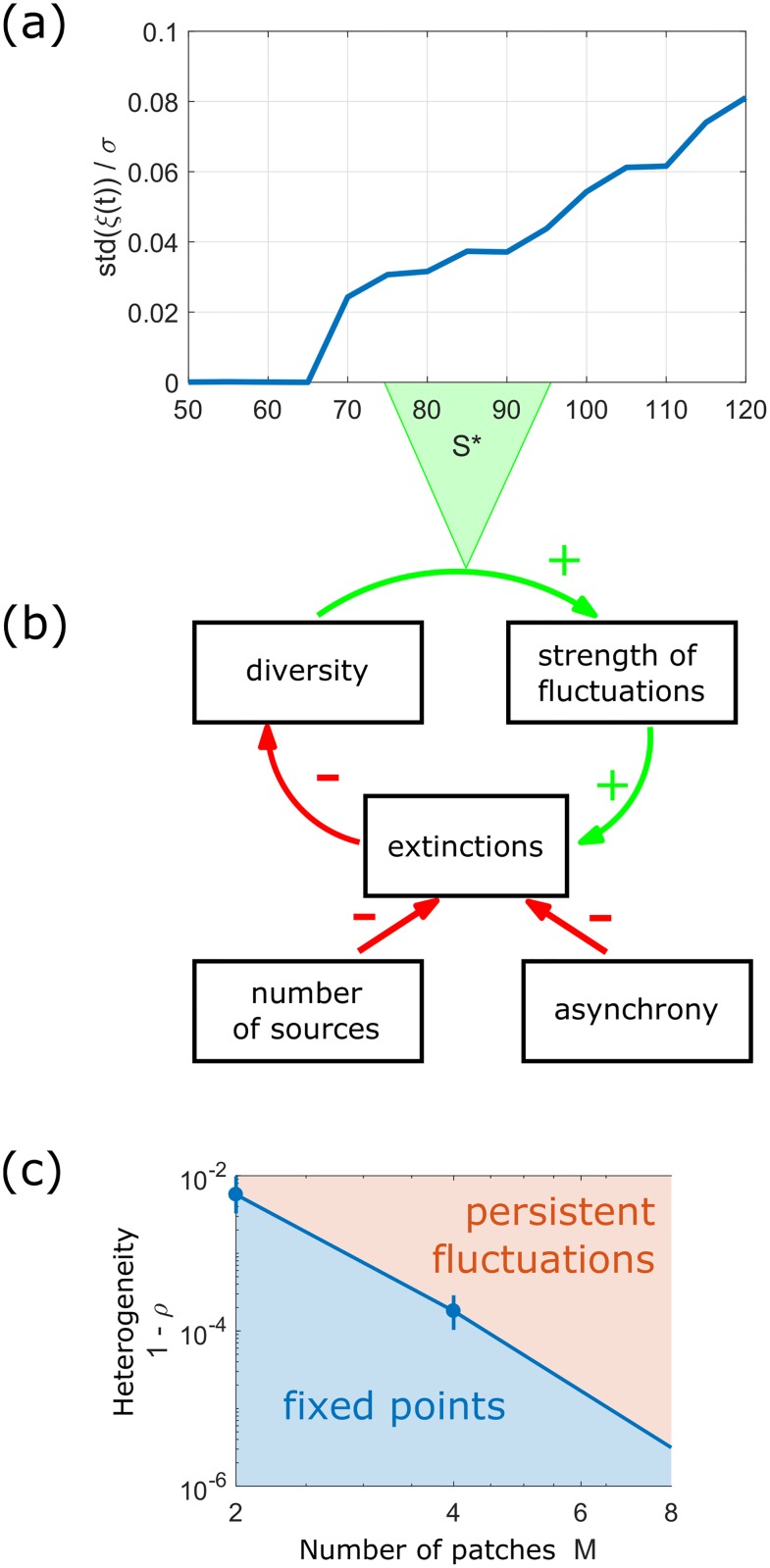
Revisiting the noise-diversity feedback cycle in the light of our theoretical framework. (a) Quantitative relationship between species diversity S*, i.e. the number of coexisting species, and strength of fluctuations std(*ξ*) for *M* = 8 (rescaled by interaction heterogeneity *σ*). (c) Patch number *M* and heterogeneity 1 − *ρ* (defined from the correlation coefficient *ρ* between interactions *A*_*ij*,*u*_ in different patches *u*) both contribute to the persistence of endogenous fluctuations by two means, shown in (b): they create source patches where a given species will tend to grow (see Fig 4), and allow the asynchrony of fluctuations in different patches. These two factors mitigate the likelihood that endogenous fluctuations will induce species extinctions and cause their own suppression.

Let us first consider a single community (*M* = 1). For a species to survive for long periods of time, it follows from DMFT that it must have positive N*, or else *N*(*t*) decays exponentially until the species goes extinct. Even if N* > 0, there is still a probability (per unit time) of extinction, which depends on N*, *N*_*c*_ and on the strength of the noise *ξ*(*t*). Following extinctions, a remaining species interacts with fewer fluctuating other species, causing the strength of the noise to decrease, see Fig 5(a), and with it the probability for extinction, see feedback loop in Fig 5. To generate Fig 5(a), abundance fluctuations were measured in simulations at precisely the same conditions but with fewer surviving species. Since extinctions become very rare at long times, this could not be done by running the simulations for longer times. Instead, species where removed from the simulation, starting with those that have the highest probability for extinction, see S1 Text (Appendix A) for details. Results are averaged over 3 runs.

We can develop an analytical treatment for very small cut-off *N*_*c*_ (large population size). In this case there is a large difference in time-scales between the short-term dynamics induced by endogenous fluctuations, and the long-term noise-diversity feedback cycle discussed above. In fact, the extinctions driving this feedback are due to rare events in which the abundance of species with a positive N* decreases below the (very small) cut-off *N*_*c*_. For a species in an isolated patch (*M* = 1), the time-scale for such an event is known [12, 13] to be of order of *τ*(1/*N*_*c*_)^*a*^ where *τ* is a characteristic time of the endogenous fluctuations, and *a* = 2N*/*W* is independent of *N*_*c*_, with *W* the amplitude of the endogenous fluctuations,

$$\begin{array}{c} W\equiv \int dtC_{\xi }(t,t^{\prime })\;. \end{array}$$

The important point here is that, although endogenous fluctuations disappear eventually, there is a clear *separation of time-scales* between typical endogenous fluctuations, that are fast and lead to a quasi-stationary dynamical state, and rare extreme fluctuations that cause extinctions and push the ecosystem into a different state.

While a single community might in principle achieve long-lasting endogenous fluctuations, this however requires unrealistically large population sizes and species number, see S1 Text (Appendix E). Migration between multiple patches substantially enhances persistence due to the spatial insurance effect [14]: species are more unlikely to go extinct because they need to disappear everywhere at once. The time scale for such an event is $\tau {\left(1/N_{c}\right)}^{Ma_{\text{eff}}}$ with $a_{\text{eff}}=2N_{\text{eff}}^{*}/W$ where $N_{\text{eff}}^{*}$ is an effective value for N* of a species across patches, an expression for which is given in S1 Text (Appendix C). This result is thus similar to the one identified above for one patch, raised to the power *M*. These results assume that *W* is finite, and that the noise acting on a species is independent between patches (asynchrony). Indeed, for moderate values of *D* and *ρ* not too close to one, simulations show that *C*_*ξ*_(*t*, *t*′) is a well-behaved function of *t* so that *W* is finite, and the correlation between patches is found to be very small, see S1 Text (Appendices B,C).

These expressions provide a quantitative description of the feedback cycle in Fig 5. Endogenous fluctuations disappear on the time scale at which species with characteristic abundance $N_{\text{eff}}^{*}$ of order one would go extinct. We must further account for the vanishing strength of the noise *W* as species disappear. This is shown in Fig 5(a), where the strength of the fluctuations is tightly linked to species diversity, and is zero at the diversity of fixed points. Hence, extinctions significantly increase *a*_eff_, reducing the chance for further extinctions.

**Fig 6:**
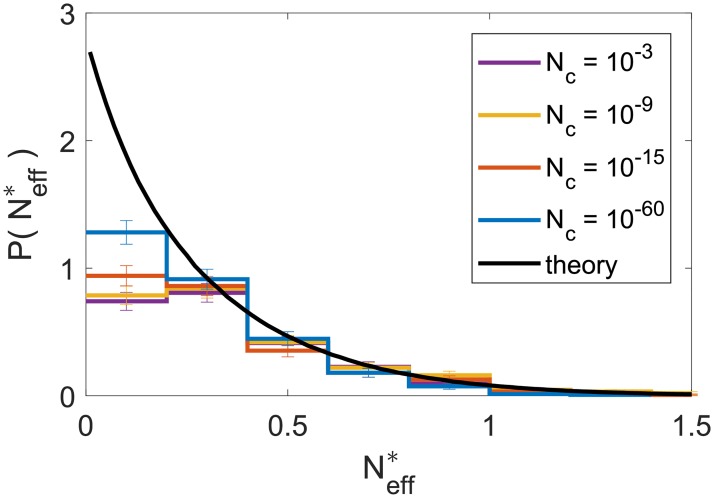
Distributions of the characteristic abundance $N_{\text{eff}}^{*}$ of surviving species, compared with the theoretical prediction for maximal diversity, showing that the lower diversity in simulations is mostly due to losing species with lowest $N_{\text{eff}}^{*}$ (leftmost bin). Reducing *N*_*c*_ (increasing population size) affects diversity mainly by allowing these “rare” species to persist.

This picture agrees well with the analytical predictions, which can be obtained for very small but positive *N*_*c*_ and *D* by DMFT. These give the distribution of $N_{\text{eff}}^{*}$, whose integral over positive $N_{\text{eff}}^{*}$ amounts to the maximal total diversity at long times. We show in Fig 6 that extinct species are generally those that have lower values of $N_{\text{eff}}^{*}$: we compare the analytically predicted distribution of $N_{\text{eff}}^{*}$ to the one observed in simulations, and see that most missing species had low values of $N_{\text{eff}}^{*}$. The difference becomes smaller for lower *N*_*c*_. Due to these differences, the obtained diversities are lower than the theoretical maximum, see Fig 3(top).

As stressed above, the asynchrony of fluctuations in different patches is crucial: it allows some species to survive with positive characteristic abundance $N_{i}^{*}$ in at least one of the patches. This leads to a higher total number of long-term persisting species, decreases the likelihood of fluctuations to small abundances, and hence increases the stability of a dynamically fluctuating state. If the migration rate *D* is too strong or *ρ* very close to one, dynamics in the different patches synchronize, quickly annulling the insurance effect. However, minor (few percent) changes in interaction coefficients or carrying capacities between patches are enough to maintain this effect, see Fig 5(bottom); we don’t need to impose coexistence artificially, e.g. by requiring that every species has at least one refuge (a patch so favorable to it that it always dominates there). These little variations in the interaction coefficients are highly plausible, as interaction strength can vary with many factors, including resource availability [25], or temperature and its influence on metabolism [26]. The heterogeneity *ρ* required to reach a fluctuating state decreases with *M*, see Fig 5(bottom).

In practice, maintaining a dynamical state seems unfeasible for only one patch, at least for reasonable values of population size *P* = 1/*N*_*c*_ and species number *S*, see discussion in S1 Text (Appendix E). Yet the combined effect of the two phenomena described above allows for very long-lived endogenous fluctuations in metacommunities, already for *M* = 2 patches.

## Discussion

Complex ecological interactions can give rise to long-lasting fluctuating states, which both require and allow the maintenance of high species diversity. This can happen under a wide range of conditions, which we have illustrated in simulated experiments, and identified through an analytical treatment based on Dynamical Mean-Field Theory. These results are robust to various modeling assumptions: our work and a parallel study by Pearce et al. [19] start from different ecological settings (fully random or antisymmetric interactions, with or without self-regulation) and build, through related methods, toward the same general conclusions, putting forward the role of spatial extension in persistent endogenous fluctuations.

While we have drawn parallels with the theory of stability and coexistence in externally-perturbed ecosystems [6, 12–14], our approach also highlights essential differences between environmentally-driven and endogenous fluctuations. We show that many-species dynamics induce feedback loops between perturbation and response, and in particular a tight relationship between fluctuation strength and species diversity, which are absent from externally-perturbed ecosystems. Moreover, while similar species can display correlated responses to environmental stochasticity [27], we expect here that their trajectories will be starkly different and unpredictable, due to high-dimensional interactions which lead to complex dynamics. The resulting picture from DMFT is that the abundance of any given species undergoes stochastic dynamics with a finite correlation time. This means that the trajectory of the species abundance cannot be predicted after a time that is large compared to the correlation time–a hallmark of chaos, also found in other models of high-dimensional systems [28]. Our theory paves the way for quantitative testing of these fingerprints of diversity-driven fluctuations in data.

In a counterpoint to classic results [8], we have shown that, while highly diverse ecosystems are unstable, they might still persist: extinctions can be avoided and biodiversity maintained, despite species abundances fluctuating over multiple orders of magnitude. We do observe a negative feedback loop, in which endogenous fluctuations cause extinctions, and eventually lead to their own disappearance as the ecosystem reaches a lower-diversity stable equilibrium. But this self-suppression of fluctuations can be mitigated by a number of factors, among which space is particularly important.

**Fig 7:**
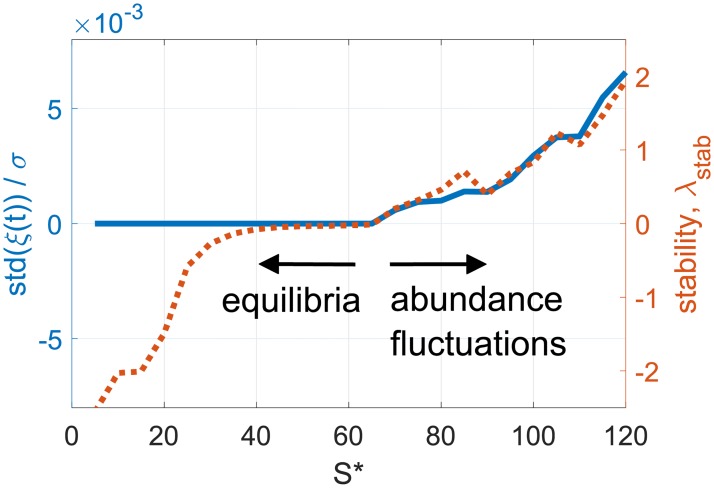
The strength of fluctuations std(*ξ*) taken from Fig 5(a) (solid line), and the linear stability λ_stab_ (dashed line). Both are measured for the same meta-community, at different diversities. Below a certain diversity, equilibria are found: abundances do not fluctuate, and the equilibrium is stable (λ_stab_ < 0). λ_stab_ increases up to this diversity, as linear stability becomes weaker. Above this diversity, abundances fluctuate in time and putative fixed points are unstable.

To make a connection between non-equilibrium dynamics and the stability of equilibria, we look at the same meta-community at different diversities S*. In Fig 7 we show the strength of abundance fluctuations taken from Fig 5(a), together with the linear stability λ_stab_ of fixed points at each diversity, defined via the spectrum of return to a fixed point, see S1 Text (Appendix A). At stable equilibria λ_stab_ < 0. Going above some diversity, stability is lost (λ_stab_ > 0) and abundance fluctuations appear, whose size is a continuously increasing function of diversity. Thus equilibria and long-lasting abundance fluctuations form part of a single continuum. The theoretical framework of DMFT generalizes tools which identify the conditions for linear stability (where λ_stab_ ≤ 0) of high-diversity equilibria [8, 11], to study high-diversity dynamically-fluctuating states.

In a single well-mixed community, we expect that persistent fluctuations might not be observed in practice: while theoretically possible, they may require unrealistic population sizes and species numbers. But spatial extension and heterogeneity can dramatically reduce these requirements, in a way that parallels the insurance effect against exogenous perturbations. When fluctuations are not synchronized across space, some patches can act as sources, from which failing populations will be rescued through migration [6, 14]. Here, we find that the existence of sources is surprisingly robust: even if there is no location where the environment is favorable to a given species, source patches can arise from interactions, and endure for long times despite the large fluctuations in species abundances. By allowing fluctuations without extinctions, spatial heterogeneity helps maintain species diversity, and thus the fluctuations themselves. This result is robust over a wide range of parameters, as it only calls for moderate values of inter-patch migration [29]: the rate *D* must be such that, over the typical time scale of abundance fluctuations, many individuals can migrate out of a patch (allowing recolonization in the absence of global extinction), while representing only a small fraction of the population in that patch.

A crucial result is that this condition suffices to ensure that synchronization between patches is absent, and that the total strength and correlation time of the noise within patches (*W* above) remain bounded for finite populations and finite migration rates between patches. This is in contrast to alternative scenarios where noise correlations decay slowly with time [30]. This result is non-trivial for endogenous fluctuations, as the existence of feedbacks (encoded in the self-consistent equations of the DMFT framework) can potentially lead to synchronization and long-time correlations in the noise. Yet we demonstrate that synchrony is avoided, both through direct simulations, and by building an analytical theory based on these assumptions, whose predictions match simulations quantitatively.

The work raises many interesting directions for future work, including the role of finite-dimensional space, where patches are only connected to their neighbors, and comparison with experiments, for example on the role of asynchrony [31].

In conclusion, non-equilibrium fluctuating states might be much more common than suggested by experiments and theory for well-mixed communities. And since these fluctuations permit the persistence of more species than could coexist at equilibrium, we might also expect significantly higher biodiversity in natural environments.

## Supporting information

S1 Text Appendices: Details of models, simulations and theory. (PDF)
