# Supplementary material for: Complex interactions can create persistent fluctuations in high-diversity ecosystems
Source: PLoS Comput Biol. 2020 May 15;16(5):e1007827. doi: 10.1371/journal.pcbi.1007827 (PMC7228057; doi:10.1371/journal.pcbi.1007827)
Supplement: S1 Text — (PDF) [file pcbi.1007827.s001.pdf]

Supporting Information (SI) for: Complex interactions can create persistent fluctuations in high-diversity ecosystems

Appendix A: Model parameters used in simulations, definitions of quantities in figures

For convenient reference, this Appendix includes the parameters for all simulations. The model is given in Eq. (1). All $B_{i,u} = 1$ and all $D_{i,uv} = d/(M-1)$. The $A_{ij,u}$ are independent for different (i,j) pairs (except in Appendix G).

In Fig. 2, the probability of $A_{ij,u}$ to be non-zero is $c = 1/8$, and the non-zero elements are sampled from a normal distribution with $\mathrm{mean}(A_{ij,u}) = 0.3$, $\mathrm{std}(A_{ij,u}) = 0.45$. The same elements $A_{ij,u}$ are non-zero across all patches u. The correlation coefficient between non-zero $A_{ij,u}$ in different patches is $\rho = \mathrm{corr}[A_{ij,u}, A_{ij,v}] = 0.95$ for $u \neq v$. (The correlation is 0.964 when interactions with $A_{ij,u} = 0$ are also counted.) The initial (pool) diversity is $S = 250$. In Fig. 2(A), $M = 1$. In Fig. 2(B), $M = 8$ patches and $d = 10^{-3}$. The cutoff is $N_c = 10^{-15}$. For each i, j, the M values $A_{ij,u=1..M}$ are drawn simultaneously from a multi-variate normal distribution with correlation matrix $C_{uv} = \rho + (1-\rho)\delta_{uv}$, using standard numerical methods (e.g., as implemented in the Matlab function `mvnrnd`).

Fig. 3(bottom), uses the same parameters as Fig. 2, but with a range of values for d, S and N_c.

Fig. 4 uses the runs shown in Fig. 2(B). Standard deviation and mean are estimated from 1601 time points during the time period $t = [10^4, 2\cdot 10^5]$. Fig. 6 uses multiple runs, with the same parameters as 2, except for $d = 10^{-4}$ and the values of N_c that are detailed in the figure legend.

Fig. 5(a) uses the same parameters as 6, and $N_c = 10^{-15}$. Fig. 5(c), shows the line where half of the runs are fixed points, and half continue to fluctuate until $t = 2\cdot 10^5$. It uses same parameters as 6, except with $D = d/(M-1) = 10^{-4}$.

In Fig. 5(a), the size of the fluctuations are calculated from $\mathrm{var}(\xi_u) = \langle \xi_u^2(t)\rangle = \sigma^2 C_{N,u}(t,t)$, with $C_{N,u}(t,t) = \langle N_u^2(t)\rangle - \lim_{t-t'\to\infty}\langle N_u(t)\, N_u(t')\rangle$. For more details on the averaging, see Appendix B, Fig. A.

Fig. 5(a) shows the strength of noise at different diversities. Extinctions beyond the time shown in simulations ($t = 2\cdot 10^5$) take extremely long times to happen, so reaching these lower diversities in simulations is unfeasible. Instead, we remove species that are most likely to go extinct. Recalling that the time to extinction is $\tau\,(1/N_c)^{2MN^*_{\mathrm{eff}}/W}$, we remove species with the lowest N_{eff}. This is done by running the system for time $\Delta t = 15\cdot 10^3$, calculating N_{eff} as for Fig. 6, and removing the 5 species with the lowest values of N_{eff}. This process is repeated. Other protocols for species removal where attempted, such as increasing N_c in time; they give similar results. The results are averaged over 3 runs, with independent sampling of interactions and initial conditions.

Fig. 7 shows the linear stability λ_{stab} of fixed points. To calculate this, the equations of motion, Eq. (1), are linearized around a fixed point $N^*_{i,u}$. This gives the matrix equation $d\vec{n}/dt = \mathbf{G}\vec{n}$, where $\vec{n}$ is a vector with one entry for each patch of a surviving species, with $n_{i,u} = N_{i,u} - N^*_{i,u}$. $\mathbf{G}$ is a square matrix, which depends on the equilibrium abundances $N^*_{i,u}$. For a state with fluctuating abundances, we use the time average of $N_{i,u}(t)$ for $N^*_{i,u}$. Let $\{\lambda_j\}$ be all the eigenvalues of $\mathbf{G}$. Then $\lambda_{\mathrm{stab}} = \max_j \Re(\lambda_j)$, where $\Re(..)$ denotes the real part. Note that a fixed point is stable if $\lambda_{\mathrm{stab}} < 0$. The results are averaged over the same 3 runs as in Fig. 5(a).

Appendix B: DMFT equations

In this section, we present the full DMFT equations, and explain how they can be reduced to the steady-state equations quoted in the main text.

We consider as a starting point equation Eq. (1). For the sake of clarity, we derive DMFT under simplifying assumptions, but the result is much more robust and could be applied to different ecology models as well as real data [1]. DMFT for ecological models has a double valency analogous to the one of mean-field theories in physics: it is at the same time an exact theory for some simple models, and a powerful approximation largely applicable to a broad range of systems. For the sake of clarity, the derivation assumes a fully connected model (all interactions are non-zero), but the results hold for any connectivity C as long as $C \gg 1$, see remark at the end of this Appendix.

The assumptions which make DMFT exact are the following: all constants $N_{i,u}(0)$, $B_{i,u}$, $D_{i,uv}$ and $A_{ij,u}$ are random variables, sampled from known distributions. More precisely:

- In each patch u and for all species i, the parameters $X^u = \{N_{i,u}(0), B_{i,u}, D_{i,uv}\}_{i=1}^S$ are drawn from a probability distribution $\mathbb{P}$ which is a product measure $\mathbb{P}_u(X^u) = \prod_{i=1}^S \mathbb{P}(X_i^u)$;
- The interaction matrix can be decomposed as $A_{ij,u} = \mu/S + \sigma/\sqrt{S}\, a_{ij,u}$. $a_{ij,u}$ are standard random variables with mean zero, variance one, and correlation:

$$\mathbb{E}\left[a_{ij,u}\, a_{kl,v}\right] = \delta_{ik}\,\delta_{jl}\,\rho_{uv}$$

where we used the Kronecker symbol δ_{ik}, and $\rho_{uv} = \rho + (1-\rho)\delta_{uv}$ is a uniform correlation ρ between patches.

With these conventions, we rewrite Eq. (1) in the following way:

$$\frac{d}{dt}N_{i,u} = N_{i,u}\left[B_{i,u} - N_{i,u} - \mu\, m_u(t) + \eta_{i,u}(t)\right] + \sum_v D_{i,uv}\left(N_{i,v} - N_{i,u}\right)$$

where $m_u(t) = S^{-1}\sum_{i=1}^{S} N_{i,u}(t)$ is the mean abundance in patch u, and $\eta_{i,u}(t) = -\sigma S^{-1/2}\sum_{j=1}^{S} a_{ij,u}\, N_{j,u}(t)$ is a species-and-patch-dependent noise.

The DMFT equation can be obtained by following Ref. [2]: in the large-S limit, it can be shown that the statistics of this multi-species deterministic process corresponds to the following one-species stochastic process, for each patch.

$$\frac{d}{dt}N_u = N_u\left[B_u - N_u - \mu\, m_u(t) + \eta_u(t)\right] + \sum_v D_{uv}\left(N_v - N_u\right) \tag{B1}$$

where $\{N_u(0),\, B_u,\, D_{uv}\}$ are sampled from the distribution $\mathbb{P}(X^u)$, $m_u(t)$ is a deterministic function, and $\eta_u(t)$ is a zero-mean Gaussian noise. The variability from one species to another becomes in the DMFT setting the randomness contained in $\{N_u(0),\, B_u,\, D_{uv}\}$ and $\eta_u(t)$.

To make this point crystal clear, let us introduce two different averages:

- $\overline{Y}$ averages over the stochastic process in Eq. (B1): over the stochastic noise η_u and over the distribution $\mathbb{P}(X^u)$;
- $\mathbb{E}_S(Y)$ denotes the statistical average over the deterministic multi-species system. $\mathbb{E}_S(Y) = \sum_{i=1}^{S} Y_i$, and therefore also includes sampling of X_i^u.

DMFT represents in terms of a stochastic process the deterministic dynamical system governing the dynamics of the S species in the ecosystem. In consequence, averages over the stochastic process coincide with average over species [2–4]: for a given observable Y: $\overline{Y} = \lim_{S\to\infty}\mathbb{E}_S(Y)$. This is analogous to the representation of the environment of an open physical system in terms of thermal noise, as it is done e.g. in the case of the Langevin equation.

The second important aspect of DMFT is *self-consistency.* This is related to the fact that the noise is induced by the dynamics of the species themselves, so its properties can be obtained from dynamical averages:

$$\begin{cases} m_u(t) = \overline{N_u(t)} \\ \langle \eta_u(t)\eta_v(t')\rangle = \sigma^2\,\rho_{uv}\,\overline{N_u(t)\,N_v(t')} \end{cases}$$

where we used a last average $\langle\cdot\rangle$ over the stochastic noise only, in order to define its covariance. Henceforth we use the notation $C^N_{uv}(t,t') = \rho_{uv}\,\overline{N_u(t)\,N_v(t')}$. These relations exactly take into account the correlations that emerge between the abundances and the interactions.

We now show how DMFT equations simplify for a time-translationally-invariant state of the system, which is in general reached after some transient time. In this state, all one-time observables become constant in time, and two-time observables become functions of the time difference only.

$$\begin{cases} m_u = \overline{N_u(t)} \\ C^N_{uv}(t,t') = C^N_{uv}(t-t') \end{cases}$$

The correlation $C^N_{uv}(t-t')$ decays at large time differences to a non-zero constant, leading to a static contribution to the noise term. In order to disentangle the static part and the time-fluctuating part of the noise, we perform the decomposition $\eta_u(t) = z_u + \xi_u(t)$ such that z_u and $\xi_u(t)$ are independent zero-mean Gaussian variables and processes verifying:

$$\langle z_u z_v\rangle = \sigma^2 \lim_{t-t'\to\infty} C^N_{uv}(t-t')$$

and subsequently $\langle \xi_u(t)\xi_v(t')\rangle = \sigma^2 C^N_{uv}(t-t') - \sigma^2 \lim_{t-t'\to\infty} C^N_{uv}(t-t')$ which vanishes for $t-t'\to\infty$.

Substituting this decomposition into Eq. (B1), we obtain:

$$\frac{d}{dt}N_u = N_u\left[N_u^* - N_u + \xi_u(t)\right] + \sum_v D_{uv}\left(N_v - N_u\right) \tag{B2}$$

where $N_u^* = 1 - \mu\, m_u + z_u$ is a Gaussian variable, whose statistics is described in Appendix D. We checked numerically that for small migration D, the noise is only correlated between patches through its static part: for $u \neq v$, $\xi_u(t)\,\xi_v(t') \ll z_u\, z_v$, as presented in Fig. A. In this case, we can write the self-consistent closure as follows:

$$\begin{cases} m_u = \lim_{t'\gg 1} \overline{N_u(t')} \\ \langle z_u z_v\rangle = \sigma^2 \lim_{t\gg t'\gg 1} C^N_{uv}(t,t') \\ \langle \xi_u(t)\xi_v(t')\rangle = \delta_{uv}\,\sigma^2\left[C^N_{uv}(t,t') - \lim_{t\gg t'\gg 1} C^N_{uv}(t,t')\right] \end{cases}$$

As explained above, DMFT can be implemented as an approximation for a large variety of systems. In this case one has to infer the average μ, the standard deviation σ of interactions, and the distribution $\mathbb{P}(X^u)$ from the data (we remind that $X^u = \{N_u(0),\, B_u,\, D_{uv}\}$) and use them as an input to define an effective model. The generalization to patch-dependent cumulants μ_u and σ_u is

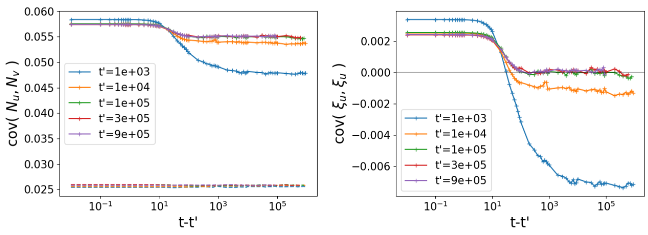

Figure A. Covariance of the abundances in distinct patches. We use the general notation $\mathrm{cov}(Y_u, Y_v) = \mathbb{E}_S[Y_u^c(t)\, Y_v^c(t')]$ and $Y_u^c(t) = Y_u(t) - \mathbb{E}_S[Y_u(t)]$. Left: In full lines we show the abundance covariance within a patch $u = v$, and across patches $u \neq v$ in dotted lines. The correlation in abundances across patches is mainly static: dotted lines are reasonably flat. In other words, the correlation of ξ_u with ξ_v for $u \neq v$ is very small. Right: The covariance in ξ is shown to reach a TTI state. It only depends on $t - t'$ after $t' = 10^5$: the colored curves collapse. In this data, 100 distinct simulations were averaged, with parameters $(S, \mu, \sigma \,|\, M, \rho, d, N_c) = (400, 10, 2 \,|\, 8, 0.95, 10^{-10}, 10^{-15})$.

quite straightforward. So is the generalization to patch-dependent correlation ρ_{uv}. With the assumptions described below, the fraction of coexisting species S^*/S is finite when S is large, so that resident diversity S^* in each community is also large.

We have derived DMFT for a completely connected set of interactions A_{ij}. A different way to obtain DMFT is considering a finite connectivity network of interactions A_{ij}, e.g. the one produced by a Erdos-Renyi random graph with average connectivity per species C or a regular random graph with connectivity C. In these cases, for each link ij one generates a random variable with average μ/C and variance σ^2/C and set it to A_{ij}. In the large connectivity limit, $C \to \infty$, each species interacts with a very large number of species and one can replace the deterministic interaction with an effective stochastic noise, as done for a completely connected lattice. Although the resulting DMFT equations are the same, the two cases are quite different: in the former a species interact with $C \ll S$ species whereas in the latter a species interacts with $C = S$ species. The equivalence of DMFT for completely connected lattices and finite connectivity ones in the $C \to \infty$ limit has been thoroughly studied in physics of disordered systems in the last twenty years [5].

In addition, the paper focuses on the case where migration connects all patches to one another. But the basic DMFT framework, Eq. (B1), is valid even if only certain patches are connected, and migration is zero otherwise. This can allow for analysis of different spatial connectivities, such as lattices reperesenting finite-dimensional space, and is an interesting direction for future work.

Appendix C: Extinction probability of a species

Here the probability of extinction of a species is presented, at the limit $N_c \ll D \ll 1$. More specifically, we assume that N_c is small compared to the typical fluctuations of the abundances. In addition, in simulations we see that it is reasonable to assume complete lack of synchrony, namely that the noise ξ_u is uncorrelated between different patches, see Appendix B, Fig. A. We will therefore assume that in the following calculation. Finally, we assume that for at least one patch, $N_u^* > 0$, otherwise the species goes quickly extinct.

Within DMFT, the problem thus becomes ones of calculating the extinction probability of a meta-population (single species), under environmental fluctuations, that are uncorrelated between the different patches. We only present the result here; a full account will be given elsewhere.

Let $x_u \equiv \ln N_u$. The equations of the DMFT, Eq. (B1), become

$$\partial_t x_u = N_u^* - e^{x_u} - \sigma\xi_u + D\sum_v \left(e^{x_v - x_u} - 1\right) . \quad \text{(C1)}$$

Here $D = d/(M-1)$. We look for a rare realization of $\{\xi_u\}$ that makes all the x_u reach $x_c = \ln N_c$, in the case when the cut-off is low, $x_c \to -\infty$. The calculation proceeds within the framework of large-deviation theory [6]. First, one defines the "action"

$$J = \frac{1}{2W}\int^{t_f} dt \sum_{u=1}^{M} \xi_u^2 \, , \quad \text{(C2)}$$

with ξ_u substituted with its value from Eq. (C1), and W defined as in the main text. Here we approximated the noise correlations by white noise, which is justified here as the extinction event takes a time which is long compared to the correlation time. We assume that D is small.

Then the mean time to the occurrence of such an event scales as $P \sim e^{J_{min}}$ with J_{min} the action J minimized over all population trajectories $\{x_u(t)\}_{u=1..M}$ that start at $t \to -\infty$ at the typical value of x_u, obtained by the zero-noise fixed point of Eq. (C1), and terminate at t_f at $x_c = \ln N_c$.

We first describe the result for $M = 1$. In this case there is only one patch, $u = 1$, with N_1^*. If $N_1^* < 0$ the species is extinct. On the other hand, if $N_1^* > 0$, then we obtain the known result [7, 8]

$$J_{\mathrm{min}} = \frac{2x_c}{W} N_1^* \, .$$

The result for all M is a generalization of this result, of the form

$$J_{\mathrm{min}} = \frac{2x_c}{W} M N_{\mathrm{eff}}^* \, .$$

To describe the calculation of N^*_{eff}, order the patches so that $N^*_1 \geq N^*_2 \geq .. \geq N^*_M$. Then there exists $1 \leq m \leq M$ such that

$$w \equiv -\sqrt{\frac{1}{m}\sum_{u=1}^{m}\left(N^*_u\right)^2} \ ,$$

and where w satisfies: $w \leq N^*_u$ for all $u \leq m$, and $w > N^*_u$ for all $u > m$. Such a partition can be shown to always exist. Then

$$N^*_{\text{eff}} \equiv \frac{1}{4M}\sum_{\{u\}_+}\frac{\left(N^*_u - w\right)^2}{w} \ .$$

The derivation will be given elsewhere. We illustrate the result by considering two cases. First, in the $M = 1$ example, since $N^*_1 > 0$, the partition is $\{u\}_+ = \{1\}$ and $\{u\}_-$ the empty set. Indeed, this gives $w = -N^*_1$, so $w \leq N^*_1$. Then $N^*_{\text{eff}} = N^*_1$ and $J_{\min} = \frac{2x_c}{W}N^*_1$, so the result for $M = 1$ is reproduced. Another simple case is when there are M patches with identical carrying capacities $N^*_{u=1..M} = N^*$. Here $\{u\}_+ = \{1, .., M\}$ and $w = -N^*$. Then $N^*_{\text{eff}} = N^*$, and $J_{\min} = \frac{2x_c}{W}MN^*_{\text{eff}} = \frac{2x_c}{W}MN^*$. This result is intuitively clear: to go extinct, the species must go extinct in all patches at once, so the probability is $P \sim \exp\left(\frac{x_c}{W}MN^*\right) \sim \left(P_1\right)^M$, where P_1 is the result for $M = 1$.

Appendix D: Diversity and stability at low migration rates

We use notations from Appendix B. Within the time-translational-invariant state:

$$\frac{1}{N_u}\frac{dN_u}{dt} = N^*_u - N_u + \xi_u(t) + \sum_{v\sim u} D_{uv}\left(\frac{N_v}{N_u} - 1\right)$$

Consider the case of low migration, $D \to 0^+$. We now develop a theory assuming that the amplitude of the endogenous fluctuations,

$$W \equiv \int dt\, C_\xi\left(t, t'\right) \ ,$$

remains finite in the limit $D \to 0^+$. Assume the species survives, i.e. there is at least one patch with $N^*_u > 0$. If $N^*_u < 0$ then $N_u = O(D)$. If $N^*_u > 0$ then $N_u = O(1)$ and therefore $\sum_{v\sim u} D_{uv}\left(\frac{N_v}{N_u} - 1\right) = O(D)$. Taking the time average of the above equation

$$0 = \overline{\frac{1}{N_u}\frac{dN_u}{dt}} = N^*_u - \overline{N_u} + O(D)$$

and therefore $\overline{N_u} = N^*_u + O(D)$.

The previous arguments lead to the conclusion that in the $D \to 0^+$ limit $\overline{N_u} = N^*_u$ if $N^*_u > 0$ and is equal to zero otherwise. In the following we provide more detail more this argument and its possible limitations. For this last equality to be valid, we need that $\sum_{v\sim u}\left(\frac{N_v}{N_u} - 1\right)$ will be finite, so that $D\sum_{v\sim u}\left(\frac{N_v}{N_u} - 1\right)$ will indeed be small. This might break if N_u can be small while some other N_v remains $O(1)$. An estimate for that proceeds by noting that the carrying capacity of patch u in the presence of other patches is larger or equal to $N^*_u - MD \simeq N^*_u$, its carrying capacity alone. If patch u fluctuates alone, then

$$\frac{dx_u}{dt} = N^*_u + \xi(t) \Rightarrow P(x) \sim e^{\frac{2N^*x}{\sigma^2 W}}$$

This gives for $\overline{1/N_u}$

$$\overline{e^{-x_u}} \sim \frac{\int_{-\infty}^0 e^{x\left(\frac{N^*_u}{W} - 1\right)}dx}{\int_{-\infty}^0 e^{x\frac{N^*_u}{W}}dx} = \frac{\frac{N^*_u}{W} + 1}{\frac{N^*_u}{W}} = 1 + \frac{W}{N^*_u}$$

For any given N^*_u this is finite. It diverges as $N^*_u \to 0$. Therefore the migration term is negligible only if $\frac{DW}{1-D} \simeq DW \ll N^*_u$. (Note that migration itself would limit N_u going below much below DN_v, which would make this term smaller.) The main approximation (or limitation) of our approach is the assumption that W remains finite in the small D limit. This is shown to hold in simulations presented in Appendix B. It breaks down if the noise develops long-lasting correlations in time. Our approximation will be nevertheless good for large $|N^*_u|$ and for weak endogenous fluctuations.

We now used the relationship discussed above between $\overline{N_u}$ and N^*_u to determine the statistics of N^*_u. We shall use the term "source" for patches where $N^*_u > 0$, and "sink" otherwise[1]. In order to understand the correlation between the sources in the different communities, we unpack N^*_u using Appendix B. Taking the time-average is equivalent to averaging over the dynamical noise ξ. Therefore, in patch u for species i, $z_{i,u} = -\sigma S^{-1/2}\sum_j a_{ij,u}\overline{N_{j,u}} = -\sigma S^{-1/2}\sum_{j,+} a_{ij,u}N^*_{j,u}$. The sum $\sum_{j,+}$ means that we only sum over $N^*_{j,u} > 0$. Here, we recall that $a_{ij,u}$ are standard random variables with mean zero, variance one, and correlation between patches:

$$\mathbb{E}\left[a_{ij,u}\, a_{kl,v}\right] = \delta_{ik}\,\delta_{jl}\,\rho_{uv}$$

where we used the Kronecker symbol δ_{ik}.

Therefore:

$$N^*_{i,u} = 1 - \mu\, m_u - \sigma S^{-1/2}\sum_{j,+} a_{ij,u}\, N^*_{j,u} \qquad \text{(D1)}$$

[1] The term "source" is used here so as to include patches (sometimes referred to as pseudo-sinks) where a species might still receive migration from patches with even larger N^*_u. But the contribution of this migration is small and not required for its persistence.

where we recall $m_u = \langle \overline{N_{i,u}} \rangle = \langle N^*_{i,u} \rangle_+$. We can now compute the different moments of the multivariate Gaussian random variable N^*_u, using equation (D1). We obtain the closure:

$$\begin{cases} \text{mean}\,[N^*_u] = 1 - \mu\, \langle N^*_{i,u} \rangle_+ \\ \text{covariance}\,[N^*_u, N^*_v] = \sigma^2 \rho_{uv}\, \langle N^*_{i,u} N^*_{i,v} \rangle_+ \end{cases}$$

When $u = v$, as $\rho_{uu} = 1$, we find the expected single community result. In particular, mean $[N^*_u]$ and variance $[N^*_u]$ do not depend on the patch u.

We numerically solve the closure in a self-consistent way: start with a guess for $\langle N^*_{i,u}\, N^*_{i,v} \rangle_+$, and then (1) Produce many samples of the vector $N^*_{u=1..M}$ and (2) calculate the next estimate for $\langle N^*_{i,u}\, N^*_{i,v} \rangle_+$, by averaging only over $N^*_{i,u}$ and $N^*_{i,v}$ that are both positive. For stability of this numerical scheme, we only replace half the samples at each iteration. We use 10^5 samples and 1000 iterations. The algorithm is always found to converge to the same solution.

Given covariance $[N^*_u, N^*_v]$, the distribution of $N^*_{i,u}$ is completely specified: it is a multivariate Gaussian in u, has the single-patch statistics of a single community, and a known covariance between patches. The solution can then also give the distribution of the number of sourcing patches.

In addition, we can compute the correlation coefficient ρ_{N^*} of the N^*_u's. We use here our simple case of a uniform correlation ρ_a between patches $\rho_{uv} = \rho_a + (1 - \rho_a)\delta_{uv}$. We introduce the notation ρ_a instead of 'ρ' in this section in order to avoid confusion with ρ_{N^*}.

$$\rho_{N^*} \equiv \frac{\text{covariance}\,[N^*_u, N^*_v]}{\text{variance}\,[N^*_u]} = \rho_a \frac{\langle N^*_{i,u}\, N^*_{i,v} \rangle_+}{\langle {N^*_{i,u}}^2 \rangle_+}$$

The results are surprising: even when $\rho_a \to 1$, the overlap between communities is not perfect ($\rho_{N^*} < 1$), so the total diversity is larger than the one in each patch. This happens *exactly at* the transition to chaos at $\sigma_c = \sqrt{2}$, see Fig. B.

On Fig. D, we compare the theory predictions to simulations. In terms of diversity, the theory appears to give an upper bound to the simulations. The difference becomes larger at higher values of σ, and for ρ_a closer to one. To look further into this difference, it is useful to study diversity as a function of the value of N^*_{eff} of each species. As shown in Fig. 6 in the main text, most of the difference in diversity is due to low values of N^*_{eff}, which are precisely the species that are more likely to go extinct, with good agreement with theory at higher values of N^*_{eff}. This is demonstrated in Fig. C, which shows that the theoretical prediction for the number of species with $N^*_{\text{eff}} > 0.2$ is closer to simulation results than the predictions for total diversity. At the moment we do not know if remaining differences are because the theoretical argument is only approximate, or whether in principle, with exceedingly low values of N_c and D, it could be approached by simulations for any σ.

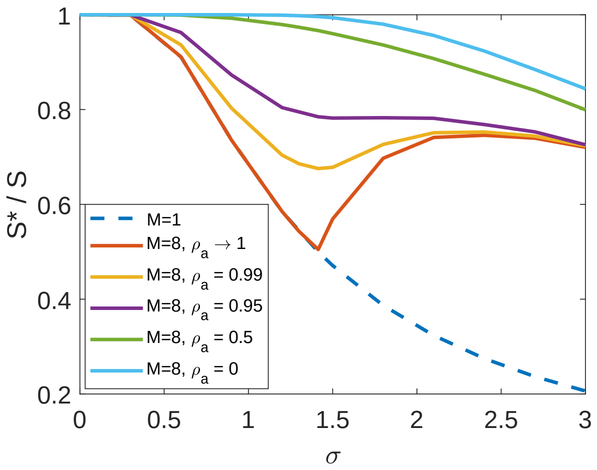

Figure B. Theoretical predictions for the diversity as a function of σ for $M = 1, 8$ patches, $\rho_a = 0, 0.5, 0.95$ and $\rho_a \to 1$.

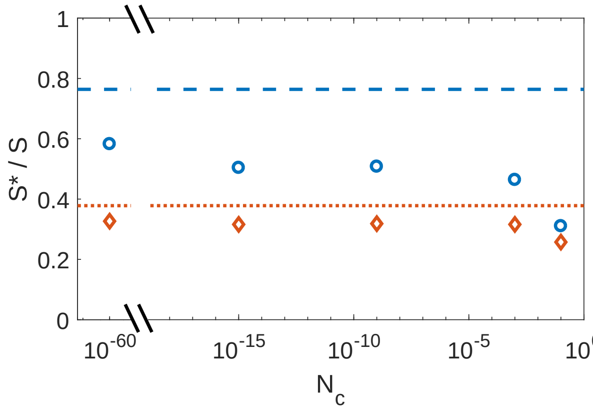

Figure C. The fraction of persistent species S^*/S (circles) is compared to theoretical bound (blue dashed line), for different values of N_c. Also shown is the fraction of species above $N^*_{\text{eff}} > 0.2$, compared to the theoretical bound for that (red dotted line), showing better agreement than for the full diversity. Simulations use the same parameters as in Fig. 6, but with a range of values for N_c (Fig. 6 corresponds to the points at $N_c = 10^{-15}$).

To find the boundary of parameter space where fixed points loose their stability and the system becomes chaotic, we look at the linear stability of persistent species. When D is small, the species that are not sourced in each patch do not affect the stability, and so the question simplifies to single patch stability, which when corr $[A_{ij}, A_{ji}] = 0$, results in $\sigma_c = \sqrt{2}$ and with $1/2$ of the species being sourced in each patch [9].

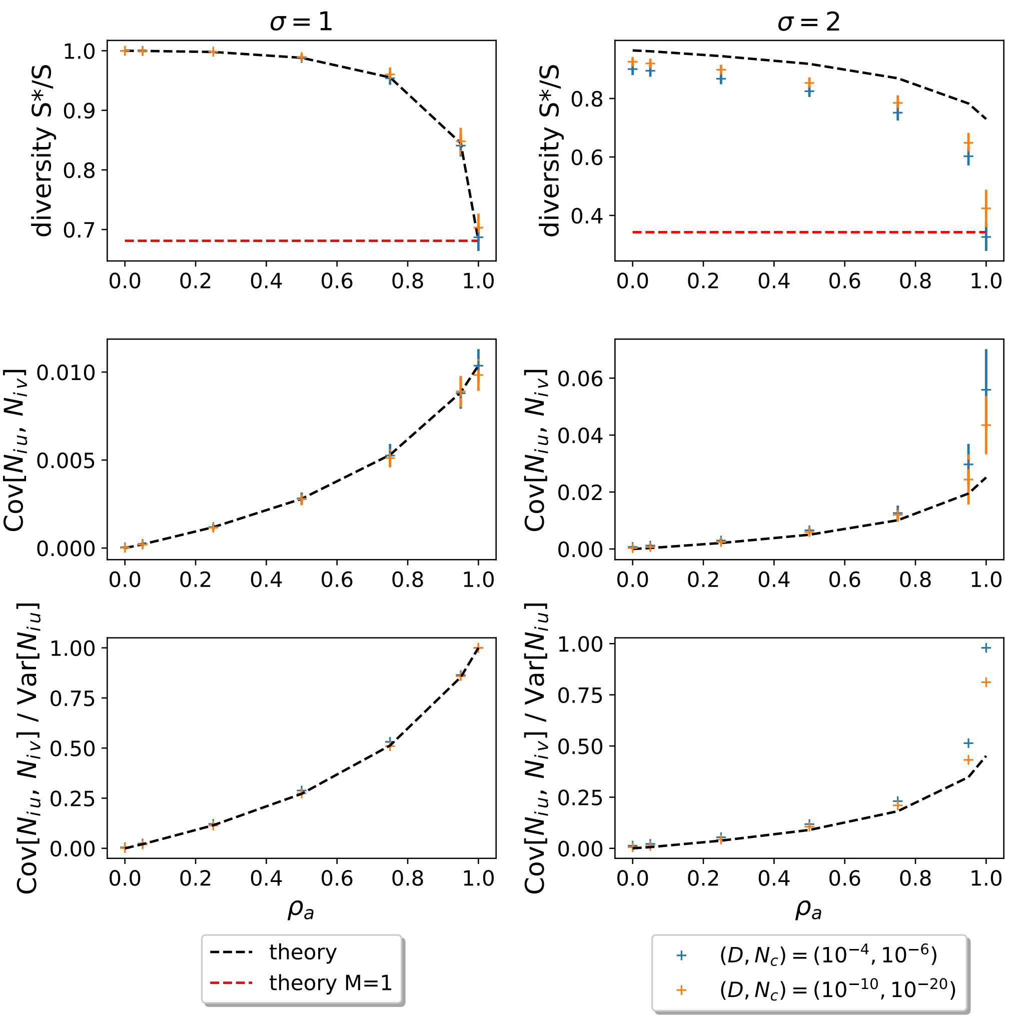

Figure D. Numerical checks of the theoretical predictions. From top to bottom, we consider three different observables: the diversity, the covariance in the abundances across distinct patches, and this covariance rescaled by the one patch variance. By varying σ, we can control the state of the system: on the left ($\sigma = 1$), we show the results for fixed points; on the right ($\sigma = 2 > \sqrt{2}$), we show the results for persistent dynamical fluctuations. In dotted lines, we plot the theory predictions, as functions of the correlation between patches' interactions ρ_a. We compare them to simulations with parameters $(S, M, \mu) = (400, 8, 10)$, and obtained by simulations run until final time $t_f = 10^4$. We eventually vary the couple (D, N_c). We use 50 distinct samples of the simulations for each combination of parameters, in order to get error bars and relevant statistics. The cut-off is implemented via patch-wise extinctions when the abundance goes below the threshold in each particular patch, in which case migration out of the patch is turned off while still allowing inward migrations.
On the left side, we can see that the theory is exact in the fixed point regime. In this regime, as $\rho_a \to 1$, the predictions are equivalent to the one patch $M = 1$ theory, as all patches are the same. In the persistent fluctuation state, the theory is only a good approximation. More precisely, the predictions become more accurate as D and N_c go to zero, as expected. In addition, the agreement gets worse when $\rho_a \to 1$, because synchronization can occur.
In the top right figure, we show that the prediction for diversity is an upper bound. In the bottom right figure, we see that indeed the prediction for ρ_n is still far from 1 when $\rho_a \to 1$, for the values of D, N_c used in the simulations.

Appendix E: Single patch ($M = 1$)

Here we show that in principle a single patch can reach and maintain a dynamically fluctuating state. However, this requires prohibitively large S, not attainable in practice. In Fig. E and Fig. F we show results of a numerical solution [2] to the DMFT equations detailed in Appendix B. At extremely low values of N_c the system appears to reach a final diversity above the May bound and, hence, to be chaotic. DMFT however describes the behavior in the $S \gg 1$ limit. When full simulations of the model in Eq. (1) are carried out at finite S, they diversity falls somewhat below the DMFT final diversity, leading to a fixed point, rather than a chaotic state, see Fig. F. This

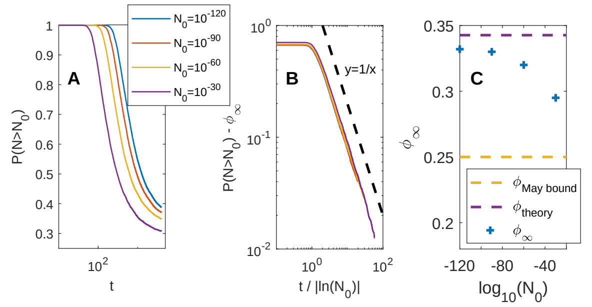

Figure E. DMFT numerics for a single patch, $M = 1$, showing that chaos is in principle possible here, although for unrealistic values of model parameters. (A) The fraction of species above different values of N_0, $P(N > N_0)$ is plotted as a function of time, for different values of N_0. (B) The curves for different N_0 collapse when $P(N > N_0) - \phi_\infty(N_0) \sim |\ln N_0| / t$. Here $\phi_\infty(N_0)$ is a fitted parameter, the extrapolated value of $P(N > N_0)$ at long times. (C) The values of $\phi_\infty(N_0)$ are well above the linear stability bound ("May bound"), and at (very) low N_0 come quite close to the theoretical maximal value for $\phi_\infty(N_0)$, predicted in Appendix D. Here $\sigma = 2, \mu = 10, N_c = 10^{-120}$.

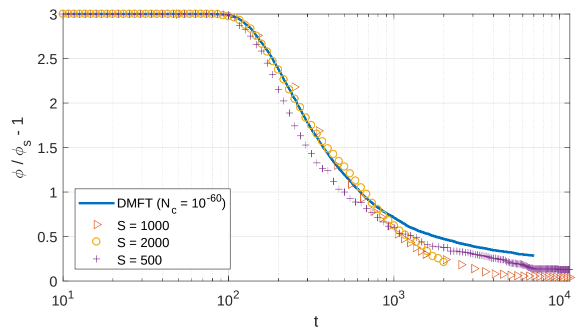

Figure F. The DMFT solution and the simulations only agree up to times $t \sim 10^3$, after which the diversity in the simulations reduces more rapidly and reaches a fixed point. This means that the convergence to the DMFT solution is slow with S.

finite-size correction to the DMFT result are important since they show that maintaining a dynamically fluctuating state for realistic values of S is not possible for $M = 1$.

Appendix F: Correlations of interactions in a pair of species

In the main text we assumed that $A_{ij,u}$ is sampled independently from $A_{ji,u}$. Here we show that the long-lived endogenous fluctuations can be found even if this assumption is relaxed. For this purpose, we consider a symmetric network of non-zero $A_{ij,u}$, namely $A_{ij,u} \neq 0$ if and only if $A_{ji,u}$. We define γ the correlation of the non-zero elements $\gamma = \text{corr}\left[A_{ij,u},\, A_{ji,u}\right]_{A_{ij,u} \neq 0}$. Fig. G

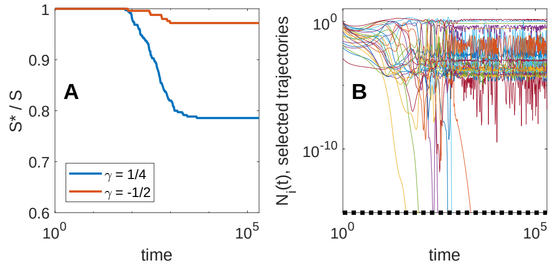

Figure G. (A) The diversity $S^*(t)/S$ for two runs with $\gamma \equiv \text{corr}[A_{ij,u}, A_{ji,u}] \neq 0$. (B) Selected trajectories of $N_{i,u}(t)$ for the run with $\gamma = 1/4$.

shows two simulations, one with $\gamma > 0$ and the other with $\gamma < 0$. In both cases the system relaxes to a long-lived state with fluctuating abundances, without further loss of diversity up to time $2 \cdot 10^5$. They are intended solely to demonstrate that conditions with $\gamma \neq 0$ exist, rather than a systematic exploration of such cases.

The parameters for the simulations (using the notation of Appendix A) are the following:

Run with positive γ: $\gamma = 1/4$, $S = 350$, $\text{mean}(A_{ij,u}) = 0.075$, $\text{std}(A_{ij,u}) = 0.175$, $c = 0.357$, $M = 8$, $d = 10^{-3}$, $\rho = 0$, $N_c = 10^{-15}$.

Run with negative γ: $\gamma = -1/2$, $S = 250$, $\text{mean}(A_{ij,u}) = 0.075$, $\text{std}(A_{ij,u}) = 0.358$, $c = 0.5$, $M = 8$, $d = 10^{-3}$, $\rho = 0$, $N_c = 10^{-15}$.

[1] Matthieu Barbier, Jean-François Arnoldi, Guy Bunin, and Michel Loreau. Generic assembly patterns in complex ecological communities. *Proceedings of the National Academy of Sciences*, 115(9):2156–2161, February 2018.

[2] F Roy, G Biroli, G Bunin, and C Cammarota. Numerical implementation of dynamical mean field theory for disordered systems: Application to the Lotka–Volterra model of ecosystems. *Journal of Physics A: Mathematical and Theoretical*, 52(48):484001, November 2019.

[3] Marc Mézard, Giorgio Parisi, and Miguel Virasoro. *Spin Glass Theory and beyond: An Introduction to the Replica Method and Its Applications*, volume 9. World Scientific Publishing Co Inc, 1987.

[4] G. Ben Arous and A. Guionnet. Symmetric Langevin spin glass dynamics. *The Annals of Probability*, 25(3):1367–1422, July 1997.

[5] Marc Mézard and Giorgio Parisi. The Bethe lattice spin glass revisited. *The European Physical Journal B-Condensed Matter and Complex Systems*, 20(2):217–233, 2001.

[6] Otso Ovaskainen and Baruch Meerson. Stochastic models of population extinction. *Trends in Ecology & Evolution*,

25(11):643–652, November 2010.

[7] Russell Lande. Risks of Population Extinction from Demographic and Environmental Stochasticity and Random Catastrophes. *The American Naturalist*, 142(6):911–927, 1993.

[8] Alex Kamenev, Baruch Meerson, and Boris Shklovskii. How Colored Environmental Noise Affects Population Extinction. *Physical Review Letters*, 101(26):268103, December 2008.

[9] Guy Bunin. Ecological communities with Lotka-Volterra dynamics. *Physical Review E*, 95(4), April 2017.
